# Firing on all cylinders: Configuring information technology around the constituents of corporate entrepreneurship to outperform in SME sector

**DOI:** 10.1371/journal.pone.0256539

**Published:** 2021-09-02

**Authors:** Nabeel Rehman, Asif Mahmood, Amir Ikram, Ayyaz Ahmad

**Affiliations:** 1 School of Accountancy & Finance, University of Lahore, Lahore, Pakistan; 2 Business Studies Department, Namal Institute, Mianwali, Pakistan; 3 Instutute of Business & Management, University of Engineering and Technology, Lahore, Pakistan; 4 Institute of Quality& Technology Management, University of the Punjab, Lahore, Pakistan; University of Salento, ITALY

## Abstract

This study has examined how small and medium enterprises (SMEs) may enhance their performance under different settings of information technology (IT) capabilities and corporate entrepreneurship (CE). Established on the dynamic capability view, the researchers have analyzed the connections between IT capabilities and CE, in addition to the performance results of SMEs. The research has explored these novel relationships by utilizing partial least square-structural equation modeling (PLS-SEM) with a data sample of 447 SMEs of the manufacturing sector in Pakistan. The findings present that IT capabilities positively influence the market and financial performance of SMEs through the mediating role of CE dimensions. The study uniquely determines the mediating role of dimensional effects of corporate entrepreneurship between IT capabilities and performance outcomes of firms. Thus, the study would enable the management of SMEs to realize the potential of IT-related CE dimensions and their use to improve firms’ performance.

## Introduction

Globalization and furious transglobal competition driven by technological developments have framed an utterly new business milieu for manufacturing organizations. In this backdrop, Small and Medium Enterprises (SMEs) contribute to achieving the growth targets of developing, emerging, and developed economies around the globe, thereby becoming mainstream economic activities [1,2]. However, these SMEs are continuously facing performance issues, predominantly in emerging economies. It is because, the drastic advancements in technology and the business environment have become more competitive [3].

The IT is a lifeline for SMEs, and it has shown to be a critical tool for improving performance and gaining a competitive advantage in the contemporary technological era [4–6]. Overall, the literature highlights that IT capabilities positively impact SME’s performance [7–9]. However, scholars have mainly targeted large and multinational firms [10,11] rather than SMEs with reference to the utilization of IT [12]. IT capability implies a company’s capability to unify and implement IT-based reserves in conjunction with other skills to improve key performance values [13].

Similarly, a major part of the literature reveals that CE is an important source for multinational enterprises and large firms to rejuvenate their businesses [14]. The literature also demonstrates that CE is an effective way for SMEs to revitalize themselves [15]. The entrepreneurial companies generally have an advantage in such an environment, considering that they are small and more flexible towards change [16]. These companies improve their strategic, structural, and procedural matters to compete in competitive environments. CE is considered the main factor in a firm’s economic development because of its vivid and positive impact on its performance and revival [17]. In fact, CE is an attitude or behavior adopted by an enterprise irrespective of its size. Furthermore, it includes not only the start-up of a new company, but also interrelated events, for instance the expansion of new services, products, competitive strategies, administrative approaches, and technologies [18,19].

Since the intervening mechanism helps improve our understanding [20]. In this relation, CE supports to explicate IT capabilities and firm performance relationship [21,22]. SMEs that use and develop IT capabilities in a dynamic business environment could support CE and ultimately improve their performance and competitiveness. Despite the extensive literature focused on IT capabilities and CE [22], few deficiencies exist. First, studies have analyzed IT capabilities at an aggregate level, while the multidimensional significance of IT abilities in theory and practice remains a mystery. Second, limited studies have focused on the multifaceted dimension of CE between IT capabilities and performance outcomes of SMEs. Third, there is a dearth of knowledge about the interrelationship between the dimensions of CE. Lastly, the researchers mainly focus on the overall (combination of market and financial) performance of the enterprise [21,23]. As both the performance indicators are different in nature [24], it is imperative to look at the impact of IT competencies on a corporation’s market and financial performance independently, as well as how they interact [25]. Furthermore, the study analyzes how these dimensions of CE and their inter-relations impact, an SME’s performance outcomes.

The literary studies emphasize the prominence of adopting the dynamic capabilities view in explaining the processes through which IT enhances a firm’s performance operating under the constantly changing business environment [9]. Because corporate entrepreneurship is regarded a dynamic capability, its basic processes have the ability to manage potential uncertainties in the business world, the current study has developed a theoretical foundation on dynamic capabilities. Similarly, innovation efforts enable businesses to compete with one another and keep one step ahead of the competition. Thus, the research contributes to the IT literature by developing a theoretical model that traces the direction from IT capabilities dimensions to performance of SME with the intervening role of CE dimensions. The study also adds to the existing literature of SMEs by unscrambling the measures of SME’s performance into financial and market performance discretely. It demonstrates that how CE dimensions affect different performance outcomes of SMEs. On a practical canvass, the findings of the study would help better identify the environment of business where considerable IT investments can be proved valuable. In addition, it would help them make informed decisions, supporting their verdicts related to the use and implementation of IT.

The paper has been organized in the following way to meet the research objectives: Section one entails a detailed background discussion. Section two reviews earlier literature as the justification for suggesting several hypotheses and is further divided into three stages: Stage I presents the relationship of IT capabilities dimensions with CE dimensions: new business venturing, innovation, proactiveness, and self-renewal. Stage II shows the interrelations of CE dimensions. Stage III presents the relationship of CE dimensions with the performance outcomes of SMEs (financial and market). The next section demonstrates the research methodology and sampling design. Section four depicts the outcomes of the analysis, and empirically examines the hypotheses developed in section two by focusing on Pakistani manufacturing firms. In the end, section five provides a detailed discussion of the results and provides future recommendations along with the study limitations.

## Literature review and hypotheses development

### Article selection

Systematic research requires examining the most relevant research work [26]. Similarly, it is imperative to make sure the quality and completeness of the articles. Accordingly, a systematic literature review was conducted [27–29]. A variety of literature was initially searched in the area of corporate entrepreneurship using multiple databases. The keywords used to explore the articles were related to dimensions of information technology, corporate entrepreneurship, as well as financial and market performance.

The early research resulted in 869 journal articles from eminent publishers such as Willey, Science Direct, SpringerLink and Emerald, published mainly during 2012–2021. Despite the conscientious effort in collating the papers, there might be some articles omitted in the list. A number of studies were reviewed, but here, the findings of only shortlisted papers have been presented for conciseness.

### STAGE I: Association between IT capabilities and CE dimensions

#### IT infrastructure flexibility and corporate entrepreneurship dimensions

IT infrastructure flexibility is defined as to what extent the IT infrastructure of a firm is modular, compatible, and scalable with the legacy systems, and is capable of addressing the multiple applications of a business [30]. IT infrastructure helps businesses to share IT-related knowledge, allowing them to engage in innovative activities while also supporting their processes and procedures [13]. This expertise reinforces the innovation process and enables the management to efficiently carry out the business tasks [31,32]. This capability also generates market equilibrium by accelerating innovative pursuits [33]; therefore, the firms need to expand IT infrastructure suppleness to drive the firm towards innovation.

IT infrastructure development also equips the firm with efficient communication, cooperation, and well-coordinated activities through inter-departmental association. IT infrastructure provides insight to identify new business ventures, and execute these ventures thereof [34]. Moreover, it helps the business managers in decision making and strategy formulation to identify and execute venturing activities [35]. It also facilitates entrepreneurial endeavors by renewing the enterprise’s ongoing needs [36], and shapes its processes by making the firm invest in restoration pursuits [37,38]. Therefore, IT infrastructure flexibility can leverage a firm’s CE activities [39].

Furthermore, data collection from diverse sources and prompt updates of consumers’ preferences are required to boost sales and develop new products [40]. In this regard, it is pertinent to mention the case of General Motors, in which the development of web-enabled tools was acquired to gather data regarding consumer preferences to design better products [41]. Such information of different stakeholders (partners and competitors) is used to absorb the effect of possible market changes and new trends. Therefore, new opportunities can be discovered using the latest IT-based tools available in the market. Hence, flexible IT infrastructure helps in adopting a proactive approach for decision-making, which is crucial for the firms to continue their entrepreneurial initiatives. Considering these arguments, we propose:

H_1a_: There is significant influence of IT infrastructure flexibility on innovation
H_1b_: There is significant influence of IT infrastructure flexibility on self-renewal
H_1c_: There is significant influence of IT infrastructure flexibility on new business venturing
H_1d_: There is significant influence of IT infrastructure flexibility on proactiveness

#### IT technical skills and corporate entrepreneurship dimensions

IT technical skills are referred to as the broad level of categorical skills that are acquired by the company’s IT workforce for IT application development. The technical abilities of IT are the ability to create IT-based applications while operating and developing products using existing technology. These include knowledge related to programming languages, operating systems, designing databases, data warehouse management, networking, and telecommunication technologies; and expertise which enables a firm to cope with the technical risks that involve IT investment [42]. Many critical aspects relating to business strategies can be managed effectively utilizing IT technical skills. If a firm promotes its technical skills, it helps achieve a greater CE level [43]. With the aid of these skills, managers of entrepreneurial firms can capitalize on new business prospects and find their way out of difficult, seemingly impossible situations [44]. These skills also provide leverage in acquiring data through speedy processes and develop an understanding of the business spectrum [13]. Furthermore, these IT skills facilitate data collection and processing, utilization and sharing of relevant technical knowledge among various working units, and play a critical role in facilitating entrepreneurial judgment regarding changing the business environment [45].

**Fig 1:**
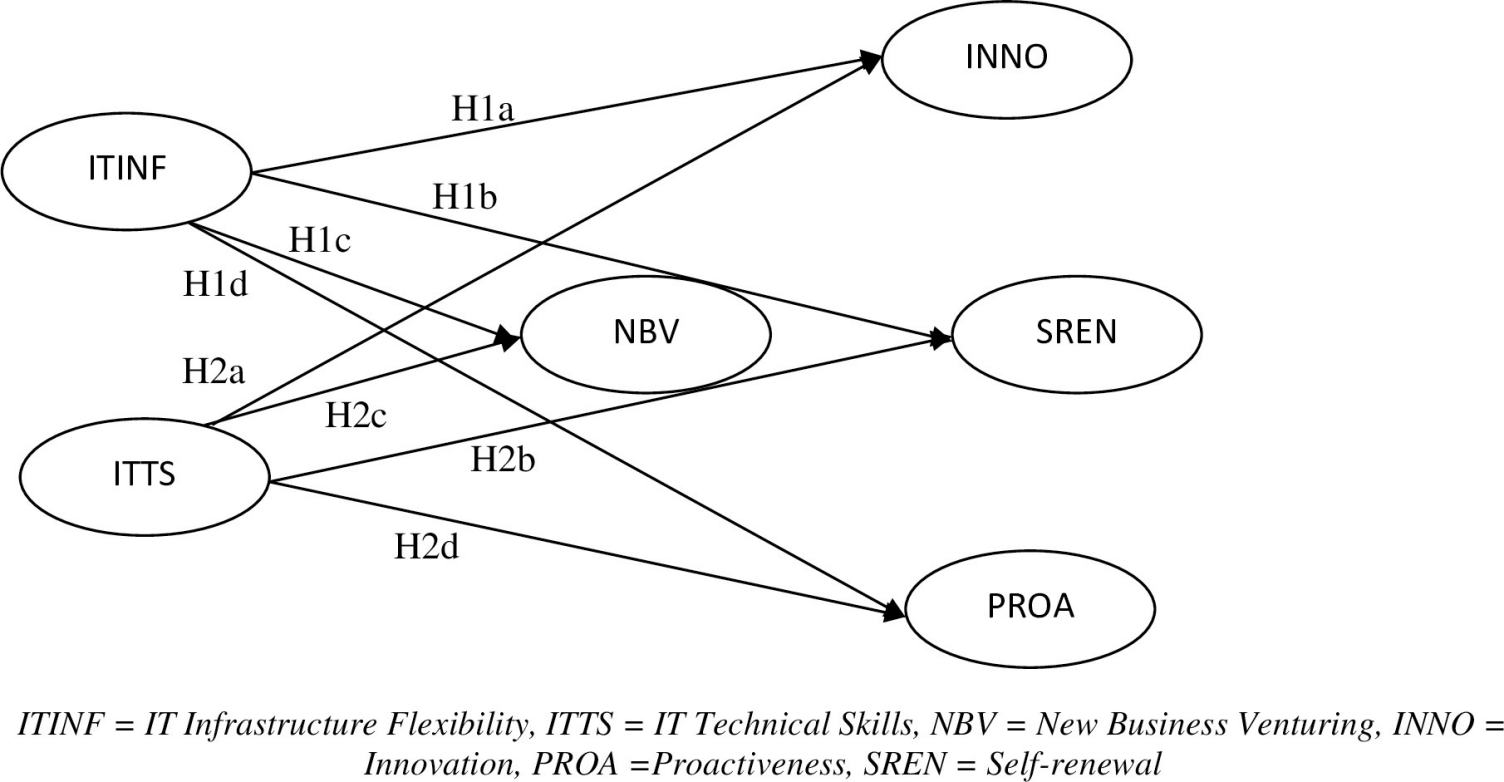
Conceptual model, Stage I. ITINF = IT Infrastructure Flexibility, ITTS = IT Technical Skills, NBV = New Business Venturing, INNO = Innovation, PROA = Proactiveness, SREN = Self-renewal.

Entrepreneurs acquire a chain of unique skills (amongst them are technological skills) that allow them to improve the innovation process and present new business opportunities in a dynamic environment [46]. Firms’ innovative technological and dynamic skills further improve the organizational innovation process and enhance the technologically preemptive behavior [47]. In addition, a group of people with a proactive attitude helps develop successful technology-based ventures [48]. Their technical skills are critical in revamping the firm for exceptional CE grip [49,50]. IT technical skills also contribute to sort issues regarding the collection and interpretation of data about the competitors and changing market trends. Such information helps initiate new business ventures [51,52]. The relationship between IT Capabilities and CE Dimensions is depicted in the conceptual model of Fig 1. Therefore, a firm’s success is eventually based on the technologically experienced group of people to support CE [53]. Consequently, we propose that:

H_2a_: IT technical skills have a significant influence on innovation
H_2b_: IT technical skills have a significant influence on self-renewal
H_2c_: IT technical skills have a significant influence on new business venturing
H_2d_: IT technical skills have a significant influence on proactiveness

### Stage II: Interrelationship between CE dimensions

#### New business venturing and innovation

The basic four aspects of corporate entrepreneurship are considered in this research article, namely, company venturing, firm innovation, proactiveness, and self-renewal. The novelty of an organization’s products is referred to as innovation. It entails the creation of new products, the enhancement of current items, and the establishment of new manufacturing techniques. The main concern is whether activities of an enterprise are novel, diverse, and unique. The new business venturing is the second aspect of corporate entrepreneurship is, which is defined as the formation of start-up within an existing business by creating a new market [54]. A firm can never bet on its current products and services to cater to long-term market conditions in response to the sheer competition in the market [55]. Customers demand innovation from firms’ offerings, and unfulfilling would cause the firms to lose their customers. This makes the customers active members in the decision-making process of a firm [56]. Therefore, each new venture or product innovation must create a new market orientation, and stimulate new technical knowledge. This is especially true for technological features focusing on new technical knowledge [57]. These processes usually result in a change in a firm’s structure, policies, and behavior. This behavior forces many firms to innovate and engage in new activities because the competitive edge attained through this process is quite high if done efficiently [58]. This also extends the process of research due to the creation of new knowledge. As a case in point, the studies of biopharmaceutical firms revealed that the establishment of such enterprises necessitates an improvement in the professional backgrounds of personnel, the nature of associations developed with external partners, the technical and industrial level of the innovations generated, and the patents attained. This showed that business ventures of biopharmaceutical companies enable innovation for the firms [59]. Thus, new business ventures contribute directly to organizational innovation. Thus, following are our hypotheses based on the aforementioned contentions:

H_3a_: New business venturing has a significant effect on innovation

#### New business venturing and proactiveness

The formation of new businesses creates new opportunities for growth and development [60]. However, it is nearly always impossible to define new business ideas in relation to the new opportunities it would create [61]. For example, outsourcing is not always straightforward, no matter how economical or lucrative. Proactive firms need to anticipate changes as they occur, and position themselves accordingly in the market, and must act before their rivals do so [62]. New businesses that add to the advantage of multiple industries stress the need for proactiveness in different environments [47]. In technological firms, the pressing need is for the company to inhibit an attitude that promotes proactiveness and promotes dynamic initiatives to respond to rapid technological changes [63]. Therefore, new business ventures are positively related to proactiveness. Given this rationale, we propose:

H_3b_: New business venturing has a significant effect on proactiveness

#### Innovation and self-renewal

Self-renewal of organizations is defined as the transformation of firms by altering their foundational concepts. Self-renewal refers to a company’s change through rethinking its primary goals. It comprises reframing corporate ideas, reformation, and the start of a system-wide transformation for originality and has strategic and radical transformational implications [64]. These changes alter the current relationships between firms or between firms and their external environment [65]. It includes the renewal of policies, business orientation, and contributes directly to the organizational change. In order to renew itself, a firm must be flexible and adaptive to a change [66]. As Håkansson and Waluszewski [67] found in their study, the world is deeply interconnected through technology, and the process of innovation increases a company’s global reach and importance. Moreover, the process of innovation contributes directly to efficiency and effectiveness and leads to strategic decision-making, specialized knowledge, and stable patterns of change and cooperation [68]. Noval ideas are built on new processes, which further enrich the process of innovation [69]. Some authors further add to the argument by stating that successful innovation procedures attract further renewal of the company, adding to its technological proficiency. The study of innovation has been researched thoroughly by academics [70,71], but there is yet to be empirical evidence of a beneficial association between innovation and self-renewal. This may be in part because the definition of technology regarding strategy change has not been developed yet. However, it may be sufficiently inferred that self-renewal is favorably and significantly related to organizational innovation. Thus, we can recommend that:

H_4_: Innovation has a significant influence on self-renewal

**Fig 2:**
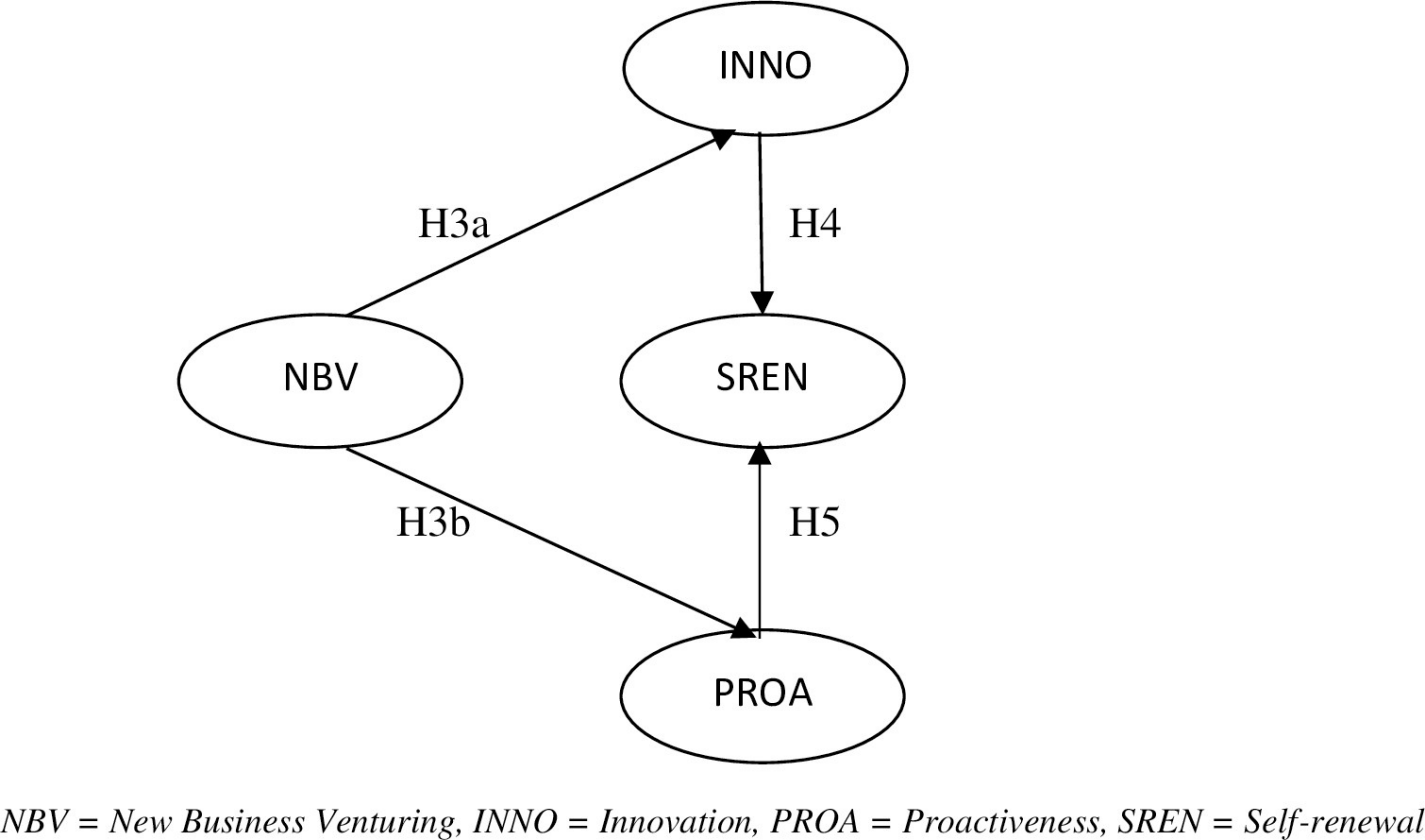
Conceptual model, Stage II. NBV = New Business Venturing, INNO = Innovation, PROA = Proactiveness, SREN = Self-renewal.

*Proactiveness and Self-renewal*. The last element of corporate entrepreneurship is proactiveness and can be defined as the actions and initiatives that were taken by the members of firms for competitive aggressiveness and risk assumptions [55]. Proactive behavior means taking control of the current circumstances or challenging the status quo. It does not mean passive acceptance of the change [72]. In the organizational context, it means a firm’s adaptability to change to be better than that of its competitors. Firms possessing a high degree of proactivity are more flexible towards change. They adapt quickly to change and their responses are more prompt [73]. Many entrepreneurs require their organizations to be proactive in retaining a competitive edge, which requires strategic flexibility [74]. Likewise, proactiveness is also seen as an enabler of a specific behavior in the firms that drive them towards flexibility [75]. The interrelationship among new business venturing, innovation, proactiveness, and self-renewal is shown in Fig 2. The strategic social change characterized by proactiveness creates self-renewal in the firms to develop new opportunities. Moreover, proactive firms manage opportunities in such a manner that offsets the negative implications of change, an advantage that conservative and rigid organizations cannot avail. These opportunities include access to newer advertising channels, supplier contracts, new sales contracts, financial capital, and participation in joint ventures [76]. But the exploitation of these opportunities demands strategic change and the renewal of policies proactively. Thus, it can be concluded here that self-renewal is linked to proactiveness positively and significantly.Thus, we can propose that:

H_5_: Proactiveness has a significant effect on self-renewal

#### STAGE II: Relationship between CE dimensions and performance outcomes

*Firm Performance and New Business Venturing*. Business venturing improves firm performance by diversifying or expanding the firm’s existing business structure. Expansion in the current structure of a business can be attained by developing start-up units in product markets linked to the product market of the parent firm. The strategic relationship between the parent firm and new venture units improves synergies by assisting in the vital resource sharing or mutual benefits of management experiences and business opportunities [77]. On the contrary, new business ventures branch out of the parent company’s product-market field when introduced to different industries. However, the introduction of new technology enhances the firm’s knowledge and helps it accomplish the market performance goals, for example, improvement in learning at different levels within the firm [78]. Thus, it is valuable for firms to gain diverse knowledge from various industries because it impels them to identify and chase growth opportunities [77].

Furthermore, business ventures adopt an effective technique suitable for their exploratory processes [79], and leads the firms to more productive advances [80]. Nevertheless, business venturing also has some disadvantages; for example, firms may face a substantial financial burden due to a high level of initial investments in business venturing. In a worst-case scenario, the income might be less than the expenses because of business venturing, which may negatively affect [81]. In addition, the investments in business venturing might be taken from other domains, for instance, from the domain of marketing or R&D, thereby deteriorating the financial performance [82]. Based on these contentions, we propose that:

H_6a_ New business venturing has a significant influence on the firm’s market performance.
H_6b_ New business venturing has a significant influence on the firm’s financial performance.

#### Innovation and firm performance

Innovation represents a mechanism of adaption, supporting firms in exploiting varying market situations such as changing customers’ needs, advancing technologies, or curbing product life cycles [83]. Innovation helps improve the existing business performance by adopting organizational processes, structure, and new product development [52]. Firms with innovative skills can straightforwardly respond to market opportunities, identify gaps and act thereof [84]. Therefore, these firms break new grounds by offering reliable services and creating new products and processes. With the development of process innovation, a firm also increases its performance by lessening the cost of production and enhancing efficiency and quality [85].

In addition, the firms that pioneer innovativeness can offer quality services and charging the desired product prices. Moreover, it helps them achieve market access control by acquiring distribution channels and marking their brands, products, and services as industry standards [52]. Such advantages provide the firms with enhanced profitability resulting from a high market share level [83]. The firms also gain a competitive advantage and improve their performance by engaging themselves in innovative activities, which support them to differentiate from the competitors [84]. Conversely, the notion of innovation may affect the performance of a firm adversely. A significant body of literature has demonstrated that poor commercialization, the uncertainty of the market, or an untimely market may result in the market breakdown of innovations [81]. Moreover, the effective implementation of innovation requires considerable resources, leading to increased risk and cost for the firm [52]. If an innovation collapses, it might prove dangerous for the enterprise’s endurance and profitability. Thus, we can propose that:

H_7a_: Innovation has a significant impact on firm’s market performance.
H_7b_: Innovation has a significant effect on firm’s financial performance.

#### Self-renewal and firm performance

A firm’s self-renewal efforts, such as redefining its mission, vision, activities reorganization, business concept, and competitiveness, are prime means for a business to adapt to changing environments. Self-renewal permits an enterprise to alter its business structure to the evolving conditions of the environment and efficiently react to these changes [54]. In fact, self-renewal efforts make a firm more insightful and, therefore, enhance its capacity to react to the external environment, i.e., opportunities and threats [86]. Self-renewal also improves the performance of an enterprise by enhancing its capacity to expand its capabilities and innovatively influence them to enhance value for shareholders [14]. Therefore, an enterprise can gain a competitive advantage by acclimating its structure to align its strategies with its environment in a better way [87,88]. However, self-renewal may also be counterproductive and may create confusion among the employees and customers. For instance, a firm may lose its customers because of its price-sensitive nature when it decides to switch from a cost leadership strategy to the strategy of differentiation [89]. Furthermore, the firm’s workforce may not make themselves out with the new strategy. It may cause low motivation among the employees that, in return, may influence the firm’s performance negatively. Thus, we conclude:

H_8a_: Self-renewal has a significant effect on a firm’s market performance
H_8b_: Self-renewal has a significant effect on a firm’s financial performance

#### Proactiveness and firm performance

Proactiveness is an attribute that foresees future market needs and problems so that enterprises become competent in capitalizing on external opportunities dynamically [55]. Proactiveness is associated with the entrepreneurial initiative processes. Management with proactive traits can visualize the essentials needed to augment the performance and growth of the enterprise [90–92], that enables to gain a competitive advantage [75]. Proactive firms are, characteristically, better equipped to create business value due to their innate capacity to respond to changing business environments in advance to seize the opportunities. In view of these arguments, we propose:

H_9a_: Proactiveness has a significant effect on the firm’s market performance
H_9b_: Proactiveness has a significant effect on the firm’s financial performance

#### Market and financial performance

**Fig 3:**
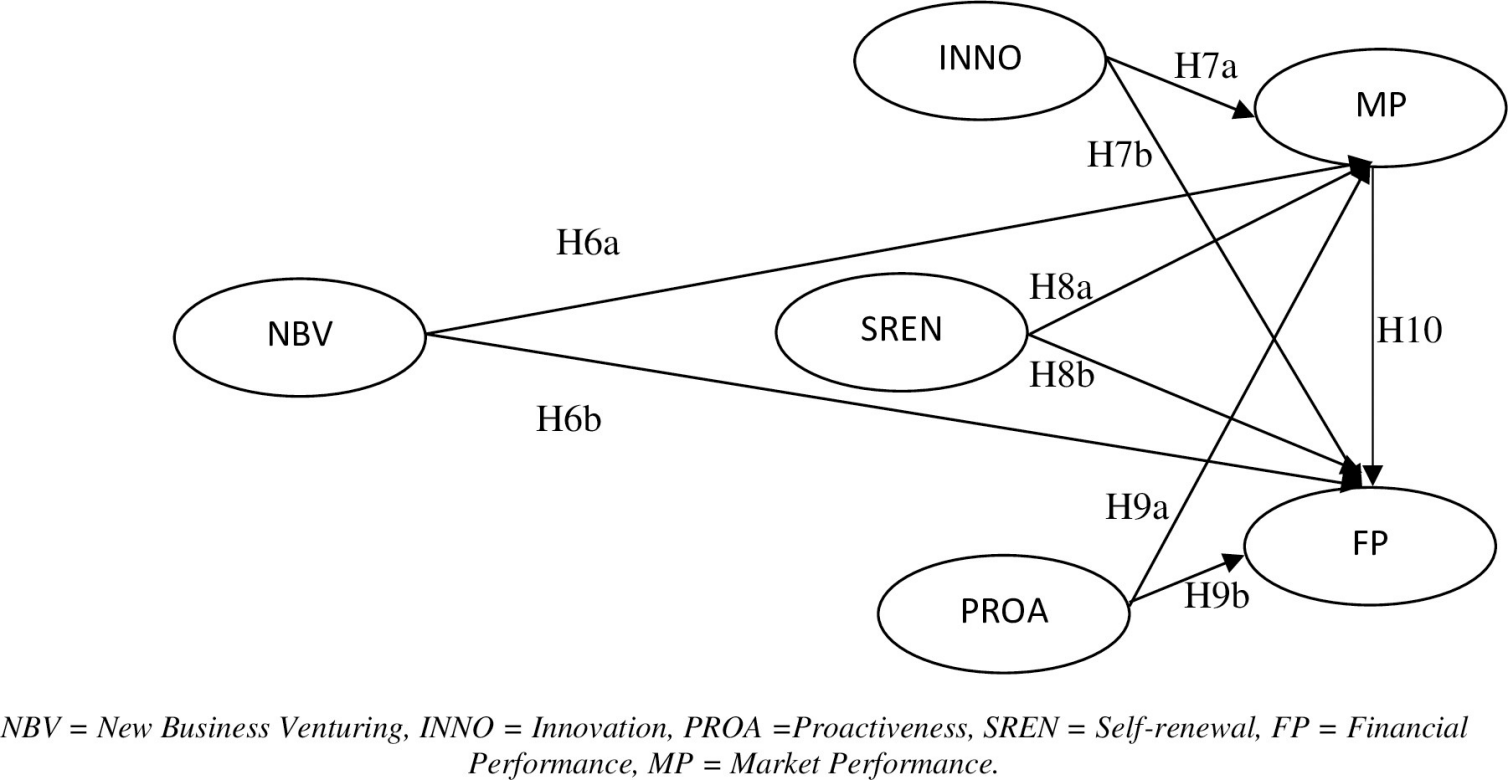
Conceptual model, Stage III. NBV = New Business Venturing, INNO = Innovation, PROA = Proactiveness, SREN = Self-renewal, FP = Financial Performance, MP = Market Performance.

Fig 3 portrays the hypotheses and interrelatedness among new business venturing, innovation, proactiveness, self-renewal, financial performance, and market performance. Marketing competency is being reinterpreted as a critical source of a firm’s financial performance in today’s customer-driven market, where the client base is considered as a key to achieving favourable financial outcomes. Subsequently, sales growth and market share may help achieve a firm’s financial goals by increasing the sales revenues and reducing the marginal unit costs, leading to significant growth in the firm’s overall profitability [3]. Thus, we suggest that:

H_10_: Market performance has a significant influence on the enterprise’s financial performance

## Method

### Sample and procedure

The small & medium enterprises (SMEs) manufacturing sector significantly contributes to the GDP and exports of Pakistan. The main manufacturing industries in Pakistan are textiles, with 21%; food industry 19%; wood & furniture 10%; metal, machinery & equipment 8%; leather 14%; sports 12%; and others 16%. There is a total of 14,722 small to medium-sized businesses in Pakistan enrolled with SMEDA (Small and Medium Enterprises Development Authority), with 8,623 of them in Punjab’s seven cities [9]. The present study chose this sector to analyze the interactions between the dimensions of IT capabilities and CE, and performance outcomes due to its contribution and importance to the economy.

As per SMEDA, the number of employees in medium-sized manufacturing firms ranges between 50 to 250. SMEDA has the core responsibility to maintain the data of SMEs and does the registration process of these firms. By following the guidelines of Lindner, et al. [93] about sample size, the size was 447 respondents selected from the database of SMEDA. The statistical power analysis demonstrates that this sample size is large enough (>150) to detect small path coefficients, improve overall estimates and standard errors, ensuring statistical power of more than 80%.

The data were collected from seven major cities of the Punjab province, namely Lahore, Faisalabad, Sialkot, Gujranwala, Gujrat, Multan, and Sheikhupura. The metropolises were selected based on their greater share (65%) of the industries in Punjab. We chose probability sampling because the population size is known, and the sampling technique used in this study is stratified random sampling, with small and medium firms serving as two ‘strata’. As a result, the sample included a proportionate number of small and medium businesses.

In an effort to decrease the non-response bias, the characteristics of businesses have been compared with the non-responding businesses. No significant difference has been found between the respondent and non-respondent businesses. Furthermore, Harman’s test has been deployed to evaluate the common method bias. We examined the common method bias by making use of Harmon’s single-factor test [94]. From the items, we retrieved the eight components having eigenvalues greater than 1. These factors accounted for 69.7% of the overall variance, while a single component accounted for only 23.4 percent. As a result, because the single component did not explain most of the total variance, it suggests that there are many factors. This means that the common method bias was not considerably influenced in the present study.

In this study, the primary respondents were the CEOs because they possess all the information from different departments. Furthermore, considering the nature of this study, CEOs are the most relevant respondents to provide the information necessary to evaluate the variables involved in our research [95]. A list containing information of top management/CEOs was formed from the enterprises enrolled with SMEDA.

The response of the construct items was collected through a 7-point Likert scale, 1 = strongly disagree and 7 = strongly agree. A total of 1800 questionnaires were distributed through e-mails and by hand with the aim to generate a high response level from the firms. The survey firms were also contacted for their services to boost the response rate further. Achieving a response rate of 27.16%, we received 133 questionnaires back through e-mails, 172 from personal contacts, and 184 through the survey firms. Thus, 489 questionnaires were received out of 1800 distributed, 42 were incomplete. Therefore, the remaining 447, completed in all respects, were taken as a final dataset. Moreover, 57% firms of the respondents were older than five years. A summary showing sample distribution by the industries has been portrayed in Table 1.

**Table 1:** Sample distribution by industry.

Industry	No. of Firms	Percentage	Questionnaire Distributed	Questionnaire Received
**Textile**	1811	21	378	101
**Leather**	1207	14	252	67
**Sports**	1034	12	216	58
**Food & beverages**	1638	19	342	88
**Metal**	690	8	144	35
**Wood & furniture**	823	10	180	42
**Others**	1380	16	288	56
**Total**	**8623**	**100**	**1800**	**447**

### Ethics statement

In compliance with local legislation and institutional regulations, no ethical review or permission was necessary for this study on human volunteers. Furthermore, the subjects gave their written consent to take part in the research.

### Measures

The study applied established measures from the existing literature as exhibited in appendix-I. IT capabilities were assessed through two dimensions: IT infrastructure flexibility and IT technical skills. IT infrastructure flexibility was measured using a four-item scale adapted from Ray, et al. [32]. The informants were asked to provide their responses regarding the connectivity, modularity, and compatibility of IT infrastructure flexibility of their firm. Whereas, a four-item scale has been used to measure IT technical skills [42]. The respondents have been asked to compare hardware operating system performance, application software performance, communication service efficiency, and program language generation with the competitors. Corporate entrepreneurship is a combination of four distinct aspects: organizational innovation, self-renewal, new business venturing and proactiveness [17,86]. The present research has adopted sixteen items to measure CE: four items for organizational innovation developed by Zahra [86], four elements for start-up venturing developed by Zahra [86], four items for proactiveness established by Knight [96], and four items for self-renewal developed by Zahra [86].

The researchers have been using market and financial perceptions relating to a firm’s management to measure the firm-related outcomes. This study assessed the performance outcomes by using both financial and market measures separately. Financial performance was assessed by the financial performance measures (ROA, ROE, and ROS) developed by Dess and Robinson Jr [97]. Market performance was assessed by sales performance, market share, and sale growth. Both financial and market performances were compared with competitors during the last three years.

### Data analysis and results

#### Descriptive statistics

The descriptive statistics for ITINF (IT Infrastructure Flexibility), ITTS (IT Technical Skills) NBV (New Business Venturing), INNO (Innovation), PROA (Proactiveness), SREN (Self-renewal), FP (Financial performance), and MP (Market Performance) have been displayed in Table 2.

**Table 2:** The descriptive statistics.

	N	Minimum	Maximum	Mean	Std. Deviation	Skewness	Kurtosis
FP	447	2.00	7.00	5.7192	.89148	1.201	−6.413
INNO	447	1.00	7.00	5.8359	.89999	−0.124	1.067
ITINF	447	1.00	7.00	5.6084	.88389	−0.415	1.856
ITTS	447	2.00	7.00	5.6771	.99414	1.086	7.196
MP	447	2.00	7.00	5.7346	0.85489	−1.044	−6.044
NBV	447	1.60	7.00	5.5167	0.74317	2.053	7.018
PROA	447	1.00	7.00	5.4872	0.88821	−1.134	−6.143
SREN	447	1.00	7.00	5.7547	0.79867	2.142	7.021

The mean of FP was 5.72, with a standard deviation of 0.89. The mean and standard deviation of INNO were 5.84 and 0.90, respectively. While the mean of ITINF was 5.61, with a standard deviation of 0.88. ITTS got a mean of 5.68 and a standard deviation of 0.99. For MP, the mean was 5.73 and the standard deviation was 0.85. The mean and standard deviation of NBV were 5.52 and 0.74, respectively. Similarly, the mean and standard deviation of PROA were 5.49 and 0.89, respectively. Finally, PROA had a mean value of 5.49 and a standard deviation of 0.74. The evaluation of Kurtosis and Skewness indicated that the data were not normally distributed because most of the values were beyond the threshold of −1.0 to +1.0 for Skewness, while within the limit (less than 10) for Kurtosis.

#### The measurement model

We used the partial least squares method to analyze our research framework using Smart PLS 3.2.6 because it facilitates the non-normality of the sample size. At first, the reliability of the constructs has been ascertained through Cronbach’s alpha and composite reliability. Table 3 represents that the construct reliability values are greater than the commonly accepted threshold value of 0.7. Then the construct validity was established through convergent and discriminant validities. It helps assess data consistency when it undergoes multiple operationalizations [98]. The convergent validity was analyzed by investigating the Average Variance Extracted (AVE) and indicator reliability of the constructs. AVE is explained by the latent construct that represents the complete variance of the indicator [99]. In this study, AVE values are above the benchmark value of 0.50, as shown in Table 3. The indicator reliability, measured through outer loadings, is above the threshold value of 0.50 [100].

**Table 3:** Reliability and loading values.

	Cronbach’s Alpha	CR	AVE	Items Loading (range)
FP	0.870	0.920	0.794	0.842–0.901
INNO	0.916	0.941	0.799	0.715–0.830
ITINF	0.820	0.881	0.649	0.742–0.865
ITTS	0.898	0.924	0.710	0.846–0.899
MP	0.885	0.929	0.813	0.826–0.881
NBV	0.907	0.935	0.782	0.869–0.915
PROA	0.925	0.947	0.816	0.867–0.916
SREN	0.919	0.943	0.805	0.865–0.889

Similarly, the discriminant validity of constructs has been ascertained through AVE values and Heterotrait-Monotrait (HTMT) ratio values [99]. The square roots of the AVE of latent constructs are compared with the appropriate inter-construct correlation estimations to calculate it. The square roots of AVE assessments are bigger than their equivalent inter-construct correlations, as seen in Table 4. As a result, the current measuring model’s discriminant validity was established.

**Table 4:** Correlation between constructs.

	FP	INNO	ITINF	ITTS	MP	NBV	PROA	SREN
**FP**	0.891							
**INNO**	0.787	0.894						
**ITINF**	0.617	0.617	0.806					
**ITTS**	0.693	0.653	0.670	0.843				
**MP**	0.787	0.765	0.583	0.679	0.902			
**NBV**	0.775	0.755	0.574	0.612	0.777	0.884		
**PROA**	0.771	0.784	0.588	0.629	0.746	0.745	0.903	
**SREN**	0.774	0.760	0.629	0.659	0.779	0.737	0.706	0.897

Note: AVE (Average Variance Extracted) values are shown as diagonal elements, and the inter-construct correlations are shown as off-diagonal.

In addition to the criterion mentioned above, we used HTMT (Heterotrait-Monotrait) ratio values to analyze the discriminant validity. As represented in Table 5, the results show that the values are within the acceptable range (threshold value 0.85). Hence, it recommends the discriminant validity of reflective constructs [101].

**Table 5:** Heterotrait-Monotrait (HTMT) ratio.

	FPn	INNO	ITINF	ITTS	MP	NBV	PROA	SREN
**FP**								
**INNO**	0.881							
**ITINF**	0.720	0.706						
**ITTS**	0.779	0.717	0.771					
**MP**	0.895	0.849	0.676	0.760				
**NBV**	0.871	0.828	0.662	0.675	0.867			
**PROA**	0.859	0.852	0.668	0.688	0.824	0.814		
**SREN**	0.864	0.827	0.718	0.722	0.862	0.807	0.765	

### Structural model

**Fig 4:**
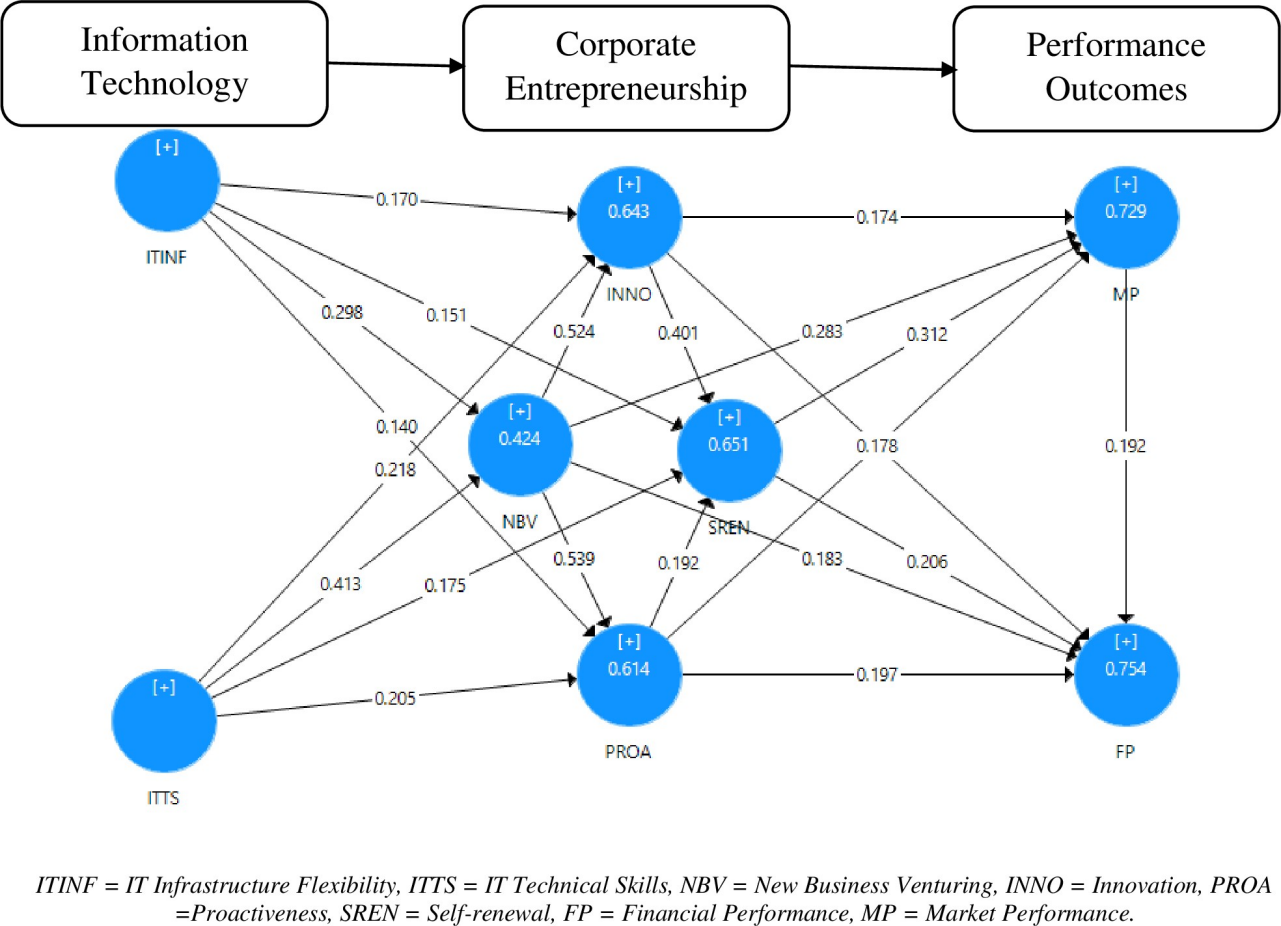
Structural model. ITINF = IT Infrastructure Flexibility, ITTS = IT Technical Skills, NBV = New Business Venturing, INNO = Innovation, PROA = Proactiveness, SREN = Self-renewal, FP = Financial Performance, MP = Market Performance.

After obtaining acceptable and appropriate results, this study moves towards analyzing research hypotheses by utilizing PLS-SEM. In stage one, the results show that ITINF significantly influence INNO, SREN, NBV, and PROA (β-value = 0.170, p-value = 0.000), (β-value = 0.151, p-value = 0.000), (β-value = 0.298, p-value = 0.005), and (β-value = 0.140, p-value = 0.000) respectively. Similarly, ITTS significantly influence INNO, SREN, NBV, and PROA (β-value = 0.218, p-value = 0.000), (β-value = 0.175, p-value = 0.003), (β-value = 0.413, p-value = 0.000), and (β-value = 0.205, p-value = 0.001) respectively. Combing stages I-III, the SEM structural model is exhibited in Fig 4.

In stage II, the results represent that NBV supports innovation and proactiveness capability of the firms (β-value = 0.524, p-value = 0.000) and (β-value = 0.539, p-value = 0.000). The results of hypothesis H_4_ demonstrate that INNO has a significant positive impact on SREN (β-value = 0.401, p-value = 0.000). Similarly, the results of hypothesis H_5_ indicate that PROA has a significant positive impact on SREN (β-value = 0.192, p-value = 0.001).

The results of the stage III demonstrate that CE dimensions have a significant positive effect on both the measures of market performance and financial performance. Based on the statistical analysis, NBV positively influences market performance and financial performance (β-value = 0.283, p-value = 0.000) and (β-value = 0.183, p-value = 0.001). In addition, INNO positively affects both the measures of performance (β-value = 0.174, p-value = 0.013) and (β-value = 0.191, p-value = 0.008). Furthermore, SREN has a positive impact on the market and financial performance (β-value = 0.312, p-value = 0.000) and (β-value = 0.206, p-value = 0.001). Similarly, PROA positively affects market performance as well as financial performance (β-value = 0.178, p-value = 0.008) and (β-value = 0.197, p-value = 0.001). Finally, the statistical results of the interrelationship of market performance and financial performance show that market performance has a positive influence on the financial performance of the enterprise (β-value = 0.192, p-value = 0.016). The empirical results of the structural model are presented in Table 6.

**Table 6:** Path coefficient.

	Paths	β-Values	T Statistics	P Values	Results
**Stage I**					
H_1a_	ITINF → INNO	0.170	3.635	0.000	Supported
H_1b_	ITINF → SREN	0.151	3.967	0.000	Supported
H_1c_	ITINF → NBV	0.298	5.095	0.000	Supported
H_1d_	ITINF → PROA	0.140	2.852	0.005	Supported
H_2a_	ITTS → INNO	0.218	4.091	0.000	Supported
H_2b_	ITTS → SREN	0.175	2.979	0.003	Supported
H_2c_	ITTS → NBV	0.413	5.973	0.000	Supported
H_2d_	ITTS → PROA	0.205	3.490	0.001	Supported
**Stage II**					
H_3a_	NBV→ INNO	0.524	9.349	0.000	Supported
H_3b_	NBV → PROA	0.539	8.113	0.000	Supported
H_4_	INNO → SREN	0.401	7.303	0.000	Supported
H_5_	PROA → SREN	0.192	3.272	0.001	Supported
**Stage III**					
H_6a_	NBV → FP	0.183	3.381	0.001	Supported
H_6b_	NBV → MP	0.283	4.097	0.000	Supported
H_7a_	INNO → FP	0.191	2.644	0.008	Supported
H_7b_	INNO → MP	0.174	2.487	0.013	Supported
H_8a_	SREN → FP	0.206	3.481	0.001	Supported
H_8b_	SREN → MP	0.312	4.889	0.000	Supported
H_9a_	PROA → FP	0.197	3.286	0.001	Supported
H_9b_	PROA → MP	0.178	2.683	0.008	Supported
H_10_	MP → FP	0.192	2.653	0.016	Supported

ITINF = IT Infrastructure Flexibility, ITTS = IT Technical Skills, NBV = New Business Venturing, INNO = Innovation, PROA = Proactiveness, SREN = Self-renewal, FP = Financial performance, MP = Market Performance.

### Mediation analysis

The research also explores the indirect effects of IT capabilities on SMEs’ performance outcomes through dimensions of CE and their interrelationships. The results of the specific indirect paths demonstrate that ITINF indirect paths to MP through NBV; INNOV and SREN, and through NBV, PROA, and SREN were significant (β-value = 0.020, p-value = 0.005), and (β-value = 0.010, p-value = 0.038) respectively. Similarly, ITINF indirect paths to FP through NBV; INNOV, were significant (β-value = 0.013, p-value = 0.007), and the path through SREN; NBV, PROA, and SREN and (β-value = 0.006, p-value = 0.051) was statistically significant but with a coefficient value.

While the results of the specific indirect path also demonstrate that ITTS indirect paths to MP through NBV; INNOV, and SREN; NBV, PROA, and SREN reach statically significant (β-value = 0.027, p-value = 0.001), and (β-value = 0.013, p-value = 0.020) respectively. Similarly, ITTS indirect paths to FP through NBV; INNOV, and SREN; NBV, PROA, and SREN reach statically significant (β-value = 0.018, p-value = 0.001), and (β-value = 0.009, p-value = 0.035) respectively.

This study also analyzes the mediating role of MP. The results of the specific indirect path demonstrate that when MP introduce as a mediator in the path from ITINF and ITTS to FP through NBV, INNOV, and SREN; NBV, PROA, and SREN could not prove to be statistically significant (β-value = 0.004, p-value = 0.075), (β-value = 0.005, p-value = 0.067), (β-value = 0.002, p-value = 0.124), and (β-value = 0.003, p-value = 0.106) respectively. These results present that MP has no mediating role in these relations.

### Goodness of Fit (GoF)

Although goodness of fit (GoF) measures generated by PLS-SEM cannot be relied upon without caution. Therefore, this study has utilized R^2^ values [102] and a diagnostic tool developed by Tenenhaus, et al. [103] to ascertain the fitness of the model. Then, GoF can be calculated by obtaining the geometric mean of AVE values and an average of R^2^ values. This study has measured GoF by following Henseler, et al. [102], as shown in Table 7. The results indicated a high level of model fitness with a value of 0.699 [104].

**Table 7:** Goodness of fit.

Constructs	AVE	R^2^
INNO	0.799	0.643
ITINF	0.649	
ITTS	0.710	
NBV	0.782	0.424
FP	0.794	0.754
PROA	0.816	0.614
MP	0.813	0.729
SERN	0.805	0.651
**Average Score**	0.771	0.635
AVE*R ^**2**^	0.489	
**GoF = √(*AVE* × *R*** ^**2**^ **)**	0.699	

In addition to R^2^ and GoF index, predictive relevance (Q^2^), referred to as predictive validity of the model, was computed to establish rigor in the modeling [105, 106]. Furthermore, the Q^2^ value provides a basis of predictive relevance other than computing the value of R^2^ magnitude as a standard of predictive accurateness. The blindfolding procedure was employed to attain the Q^2^ values of the endogenous constructs. Table 8 shows Q^2^ values are greater than 0.35 to establish a large predictive relevance.

**Table 8:** Construct crossvalidated redundancy.

	SSO	SSE	Q² (= 1-SSE/SSO)
**FP**	1,341.000	585.411	0.563
**INNO**	1,788.000	928.806	0.481
**ITINF**	1,788.000	1,788.000	
**ITTS**	2,235.000	2,235.000	
**MP**	1,341.000	591.606	0.559
**NBV**	1,788.000	1,236.002	0.309
**PROA**	1,788.000	952.344	0.467
**SREN**	1,788.000	912.939	0.489

## Discussion

In the recent period, many scholars have shown their interest in exploring the impact of CE on other constructs [7, 21, 107]. However, there exists a dire need to explain the individual impact of CE dimensions on performance-related outcomes. Considering the recommendations from the literature, the authors have carried out the research to elaborate the underlying mechanism that maps indirect paths leading to the firm’s performance from IT capabilities (IT technical skills and IT infrastructure flexibility) through the intervention of CE dimensions. It has been established through the results that IT capability dimensions (IT technical skills and IT infrastructure flexibility) have significant positive effects on the dimensions of CE, which, in turn, contributes towards the enhancement of the financial and market performance of the firm.

H_1a_— H_1d_ hypothesized that IT infrastructure flexibility significantly affects innovation, proactiveness, new business venturing and self-renewal. The empirical findings as well as previous studies supported these hypotheses (β-value = 0.170, p-value = 0.000; β-value = 0.151, p-value = 0.000; β-value = 0.298, p-value = 0.000; β-value = 0.140, p-value = 0.005). IT infrastructure facilitates the firms to share IT-related knowledge, enabling them to exercise innovation activities and support their processes and procedures [13]. This capability strengthens the innovation process and enables the management to efficiently carry out core activities of the business [31,32]. It also generates market equilibrium by accelerating innovative activities [33]. Moreover, it helps the business managers in decision making and strategy formulation identify and execute venturing activities [35]. It also facilitates a firm’s entrepreneurial activities by renewing its ongoing needs [36] and shaping its processes by investing in renewal endeavors [37,38]. Similarly, IT infrastructure provides insight to identify new business ventures and execute these ventures thereof [34]. Hence, compliant IT infrastructure helps in adopting a proactive approach for decision-making.

H_2a_— H_2d_ hypothesized that IT technical skills significantly affect innovation, proactiveness, self-renewal, new business venturing. The results are in favor of these arguments (β-value = 0.140, p-value = 0.005; β-value = 0.218, p-value = 0.000; β-value = 0.413, p-value = 0.000; β-value = 0.205, p-value = 0.001). It is because when a firm promotes its technical skills, it helps achieve a greater CE level [43]. With the aid of these skills, managers of firms are in a good position to identify new business opportunities and find their way out of difficult, seemingly impossible situations [44]. Firms’ innovative technological and dynamic skills further improve the organizational innovation process and enhance the technologically proactive attitude [47]. IT technical skills also contribute to sort issues regarding the collection and interpretation of data about the competitors and changing market trends. Such information helps initiate new business ventures [51,52].

H_3a_ and H_3b_ state that new business venturing has a significant effect on innovation and proactiveness. The findings substantiate these hypotheses (β-value = 0.524, p-value = 0.000; β-value = 0.539, p-value = 0.000). It means each new venture or product innovation must create a new market orientation and stimulate new technical knowledge. This is especially true for technological features focusing on new technical knowledge [57]. Moreover, new businesses that add to the advantage of multiple industries stress the need for proactiveness in different environments [47].

H_4_ sets out that innovation has a significant effect on self-renewal. The results confirmed this hypothesis (β-value = 0.401, p-value = 0.000).

H_5_ describes that proactiveness has a significant effect on self-renewal. The result (β-value = 0.192, p-value = 0.001) is in congruence with this assumption. In the literature, proactiveness has also been seen as an enabler of a specific behavior in the firms that drive them towards flexibility [75].

H_6a_ and H_6b_ assert that new business venturing significantly affects the firm’s market performance and financial performance. The findings (β-value = 0.183, p-value = 0.001; β-value = 0.283, p-value = 0.000) corroborate these claims. Similar results have been reported in the previous study [77].

H_7a_ and H_7b_ posit that innovation has a significant effect on a firm’s market and financial performance. The results (β-value = 0.191, p-value = 0.008; β-value = 0.174, p-value = 0.013) and the earlier research work also suggest that with the development of process innovation, a firm increases its performance by lowering the cost of production and enhancing efficiency and quality [85]. Moreover, it helps them achieve market access control by acquiring channels of distribution and marking their brands, products, and services as industry standards [52].

The results (β-value = 0.191, p-value = 0.008; β-value = 0.174, p-value = 0.013) confirmed H_7a_ and H_7b_ that innovation has a significant effect on firm’s market and financial performance. Firms with innovative skills can straightforwardly respond to market opportunities, identify gaps and act thereof [84]. With the development of process innovation, a firm also increases its performance by reducing the cost of production and improving efficiency and quality [85].

H_8a_ and H_8b_ assert that self-renewal significantly affects a firm’s market and financial performance. The findings confirm these assertions (β-value = 0.206, p-value = 0.001; β-value = 0.312, p-value = 0.000). In fact, self-renewal efforts make a firm more insightful and, therefore, enhance its capacity to react to the external environment, i.e., opportunities and threats [86].

H_9a_ and H_9b_ further state that proactiveness significantly affects the firm’s market and financial performance. The results validated these hypotheses (β-value = 0.197, p-value = 0.001; β-value = 0.178, p-value = 0.008). Management with proactive traits can visualize the essentials needed to enhance performance and growth [90–92].

Finally, H_10_ assumes that market performance has a significant effect on the firm’s financial performance. The result (β-value = 0.192, p-value = 0.016) confirms this assumption. Marketing competency is recognized as an essential source of a firm’s financial performance in today’s customer-driven market, where the client base is considered a key to achieving positive financial outcomes.

However, when this study statistically analyzes the mediating role of MP in the path from ITINF and ITTS to FP through ‘NBV→ INNOV→ SREN→MP’ and ‘NBV→ PROA → SREN→MP’, surprisingly the impact of ITINF and ITTS on FP became insignificant, indicating that MP has no mediating role in these relationships (β-value = 0.004, p-value = 0.075; β-value = 0.005, p-value = 0.067; β-value = 0.002, p-value = 0.124, and β-value = 0.003, p-value = 0.106) respectively. The reason behind the insignificant relationship is probably that SMEs pay less attention to market performance than financial performance. Another reason for the insignificance of mediation paths could be that SMEs often face difficulty attaining market knowledge. This lack of knowledge related to current market trends results in the failure of proactive activities. When SMEs are facing critical times, the policymakers should be very conscious while providing their knowledge support. Considering the current business environment, witnessing uncertain industries, and rapid technological changes, the firms have made themselves smarter and efficient enough to analyze how to respond when they see an opportunity. It makes it indispensable for firms to make thoughtful decisions while undertaking market structure.

### Implications

By proposing and elaborating different direct as well as indirect mediation paths, this study proved to be the first academic effort that emphasizes the role of IT capabilities in a firm’s performance through the mediation of CE dimensions. The research contributes to the literature by elaborating IT capabilities and CE from a perspective of their dimensional role. On a managerial front, this study would enable the management of SMEs to realize the potential of IT-related CE dimensions and their use to enhance firm performance. Consequently, the CEOs and managers would find themselves better positioned to foresee their business environment and make the right entrepreneurial investment decisions. Along with the mediation of innovation, the impact of IT infrastructure flexibility on performance firms supports carrying out their activities, particularly innovation activities, at all levels of organization. In this manner, the effective usage of IT resources would improve the flexibility of IT infrastructure, and this has mostly shown a positive impact on new product development and adaption of new processes by SMEs, which subsequently enhances its productivity [108]. Similarly, the influence of IT infrastructure flexibility on performance as a result of strategic renewal also benefits the varied activities of businesses. Therefore, intangible assets like CE dimensions and IT capabilities must be given higher importance by the businesses [108], and strategies should be made for better business outcomes by considering dynamic capabilities. Since this study has been conducted in Pakistan, which is an emerging economy that shares some similarities with the developing countries- these findings can also benefit the SME sector of these countries.

## Conclusion

This study has examined how small and medium enterprises (SMEs) may augment their performance under different settings of information technology (IT) competencies and corporate entrepreneurship (CE). Established on the dynamic capability view, the researchers analyzed the interactions between the dimensions of IT capabilities and CE and the performance outcomes of small and medium enterprises (SMEs). The research has explored these novel relationships by utilizing partial least square-structural equation modeling (PLS-SEM) with a data sample of 447 SMEs of the manufacturing sector in Pakistan. The results obtained through the analysis align with the previous empirical evidence [90–92].

The study would enable the management of SMEs to capitalize on the potential of IT-related CE dimensions and their use to improve a firm’s performance.

### Limitations and future recommendations

No study can go without limitations, and this is one of them. Our research design was cross-sectional. Although the survey respondents- managers and CEOs had adequate business knowledge and full awareness of IT-related issues of their organizations, however, the proposed framework should also be assessed through a longitudinal research design for more profound insight. Furthermore, the model of the study can be used for follow-up qualitative research as a point of departure and following a constructivist approach, whereby the IT-related CE dimensions are considered a "make-in" performance variable rather than a "maker-of" performance variable.

This research may further be extended to other Asian regions to increase the generalizability of the results and analyze the cultural and location-based differences in SMEs. Though SMEs in different regions of Asia may not necessarily exhibit other traits in terms of culture, practices, and institutional settings, such a study is still imperative to validate the findings of this study. IT capability-related research may be extended by introducing other dynamic capabilities as mediators along with their dimensional role. Besides, second-order dynamic capabilities may be explored together with other IT-related constructs because this would open an essential avenue for the researchers relating to CE and IT capabilities in SMEs.

## Supporting information

S1 Dataset (RAR)
